# Survival Outcomes After Cytoreductive Surgery with Hyperthermic Intraperitoneal Chemotherapy in Patients with Synchronous Versus Metachronous Onset of Peritoneal Metastases of Colorectal Carcinoma

**DOI:** 10.1245/s10434-022-11805-9

**Published:** 2022-05-05

**Authors:** Michelle V. Dietz, Job P. van Kooten, Ibrahim Said, Alexandra R. M. Brandt-Kerkhof, Cornelis Verhoef, Andreas J. A. Bremers, Johannes H. W. de Wilt, Philip R. de Reuver, Eva V. E. Madsen

**Affiliations:** 1 Department of Surgical Oncology, Erasmus MC Cancer Institute, Rotterdam, The Netherlands; 2 Department of Surgery, Radboud University Medical Center, Nijmegen, The Netherlands

## Abstract

**Background:**

Cytoreductive surgery (CRS) with hyperthermic intraperitoneal chemotherapy (HIPEC) is a treatment option for peritoneal metastases (PM) from colorectal carcinoma (CRC). Because of considerable morbidity, optimal patient selection is essential. This study was designed to determine the impact of the onset of PM (synchronous vs. metachronous) on survival outcomes after CRS-HIPEC.

**Methods:**

Patients undergoing CRS-HIPEC for colorectal PM in two academic centers in the Netherlands between 2010 and 2020 were eligible for inclusion. Patients were classified as synchronous (s-PM, i.e., diagnosis at time of presentation, staging, or primary surgery) or metachronous onset (m-PM, i.e., diagnosis during follow-up) of colorectal PM. Survival outcomes were compared between groups by Kaplan–Meier survival and Cox regression analyses.

**Results:**

Of 390 included patients, 179 (45.9%) had synchronous onset of colorectal PM. These patients more often presented with higher TN-stage and poor differentiation/signet cell histology. Treatment with perioperative chemotherapy was more common in s-PM patients. m-PM patients experienced more serious postoperative complications (Clavien-Dindo ≥ III). There was no significant difference in disease-free survival (DFS) between s-PM (median 9 months, interquartile range [IQR] 5–15) and m-PM patients (median 8 months, IQR 5–17). Overall survival (OS) was significantly shorter for s-PM (median 28 months, IQR 11–48) versus m-PM patients (median 33 months, IQR 18–66, *p* = 0.049). Synchronous onset of PM was not independently associated with OS in a multivariable analysis.

**Conclusions:**

Synchronous onset of colorectal PM was associated with poor tumor characteristics and more advanced disease, but was not an independent predictor of survival outcomes after CRS-HIPEC.

Approximately 4-6% of the patients with colorectal carcinoma (CRC) present with peritoneal metastases (PM) at the time of primary diagnosis (synchronous onset; s-PM).^1–3^ Another 4-6% of the CRC patients will develop PM during follow-up (metachronous onset; m-PM). Patients with colorectal PM have a poor prognosis with a median survival of about 16 months in patients treated with systemic chemotherapy.^4^ Selected patients might gain survival benefit from cytoreductive surgery (CRS) combined with intraoperative hyperthermic intraperitoneal chemotherapy (HIPEC). This treatment results in median disease-free survival (DFS) and overall survival (OS) up to 20 and 41 months, respectively.^5–10^

Due to the extent of this treatment, CRS-HIPEC is associated with severe postoperative morbidity. It is essential to select patients who will most likely benefit from this treatment. Previous studies have identified multiple factors as predictors of survival after CRS-HIPEC. Important prognostic factors are the peritoneal cancer index (PCI) and completeness of cytoreduction (CCR).^7,11,12^ However, CCR is determined intraoperatively and thus has no value in preoperative patient selection. The PCI can be estimated preoperatively by radiological assessment and/or diagnostic laparoscopy but is commonly underestimated.^13^ Hence, there is a need to identify prognostic factors that could be used in preoperative patient selection.

For colorectal liver metastases (CRLM), several studies proposed the timing of onset as a predictor of survival.^14–16^ A recent study in metastatic CRC patients reported impaired survival in patients with synchronous onset of these metastases.^17^ A few groups have published conflicting data on the impact of s-PM versus m-PM on survival outcomes in patients undergoing CRS-HIPEC (Table 1).^1,12,18–20^ The purpose of this retrospective multicenter study was to determine the prognostic value of time of onset of colorectal PM in patients undergoing CRS-HIPEC. We hypothesize that the synchronous onset of colorectal PM is a negative prognostic factor. More information contributes to a better estimation of the prognosis and could aid in shared decision making.

**Table 1:** Previous studies on the impact of the onset of PM on survival outcomes after CRS-HIPEC for PM from CRC

Study	No. patients	s-PM (%)	DFS s-PM^a^ (months)	DFS m-PM^a^ (months)	*p* value	OS s-PM^a^ (months)	OS m-PM^a^ (months)	*p* value
Hentzen et al. 2019^17^	433	53.3	15.0	11.0	< 0.001	34.0	33.0	0.819
Wong et al. 2020^18^	102	19.6	13.1	9.5	0.917	26.9	45.2	0.025
Bakkers et al. 2021^19^	88	38.6	14.1	21.5	0.094	35.8	37.8	0.553

^a^Median

## Methods

### Study Population and Definitions

Patients who underwent a complete CRS-HIPEC procedure for colorectal PM in the Erasmus Medical Center in Rotterdam between March 2014 and June 2020 and the Radboud University Medical Center in Nijmegen between March 2010 and October 2020 were eligible for inclusion. Patients with appendiceal carcinomas or without histologically proven PM were excluded. Relevant patient and disease-related characteristics, operation details, postoperative, and survival outcomes were obtained from a prospectively maintained database.

Synchronous onset of PM (s-PM) was defined as a diagnosis of colorectal PM at the time of presentation, during routine staging, or at primary surgery. If colorectal PM were diagnosed in the follow-up period, the patients were stratified in the metachronous onset (m-PM) group. The disease-free interval (DFI) was defined as the time between diagnosis of the primary tumor and the diagnosis of the PM. A cutoff value of 12 months was used to stratify the DFI as short or long. The primary outcomes of this study were DFS and OS. DFS was defined as the time interval in months between CRS-HIPEC and date of recurrence or date of last follow-up visit in censored cases. OS was defined as the time interval in months between CRS-HIPEC and date of death or date of the last update of survival status in censored cases. Information on survival status was obtained from the national civil registry, when not available in the electronic patient file.

### Preoperative Course

After referral for CRS-HIPEC, all patients were preoperatively screened. Dedicated radiologists reviewed preoperative CT scans to determine the extent of the disease. If possible, patients underwent diagnostic laparoscopy (DLS). The peritoneal cancer index (PCI) was recorded according to Jacquet and Sugarbaker.^21^ Patients were eligible for CRS-HIPEC if they were fit for major surgery and had an estimated PCI below 20 without extra-abdominal metastasis. The presence of liver metastases was no definite contra-indication for CRS-HIPEC.

### Perioperative Course

CRS-HIPEC procedures were performed by a specialized surgical team, in accordance with the Dutch CRS-HIPEC protocol.^22,23^ After median laparotomy, PCI was determined. CRS was performed when the PCI score was below 20 points and/or the surgeons presumed the PM resectable.

Patients were postoperatively treated following standard of care for CRS-HIPEC procedures. The Clavien–Dindo classification of surgical complications was used to classify postoperative complications.^24^ Severe postoperative complications were defined as Clavien–Dindo grade III or higher (i.e., reintervention, prolonged ICU stay/readmission to ICU, or treatment-related death). If a patient had multiple complications, the highest Clavien–Dindo grade was registered. The postoperative period was defined as the 30 days after CRS-HIPEC or the duration of the entire hospital stay when exceeding 30 days.

### Follow-Up

Follow-up was performed in the outpatient clinic. In the Erasmus Medical Center Carcino-Embryonal Antigen (CEA) was determined every three months and a CT scan was made every six months, or in case of rising CEA levels, during the first 2 years of follow-up. When patients were disease-free after 2 years, CEA was determined every 6 months and a CT scan was performed every 12 months, or in case of rising CEA levels. Follow-up was completed after a disease-free interval of 5 years following CRS-HIPEC. In the Radboud University Medical Center, CEA measurements and CT scans were performed every 6 months during the 5 years of follow-up. In both centers, an additional CT scan was performed in case of suspicion of recurrent disease.

### Statistical Analysis

Continuous variables were presented as median with interquartile range (IQR). Categorical variables were presented as counts with percentages. Continuous variables were compared between s-PM and m-PM patients using a Mann-Whitney *U* test. Categorical variables were compared using the Chi-square test or Fisher’s exact test if less than five events occurred. The Kaplan–Meier method was used to estimate the median OS and DFS. To compare OS and DFS between the groups, the log-rank test was used. To determine predictive factors for OS and DFS multivariable cox regression analyses with backward selection were performed. The variables age, ASA (American Society of Anesthesiologists) score, primary tumor differentiation, lymph node status, PCI, CCR score, and postoperative complications were entered in the model, as these have shown prognostic value in earlier studies.^7,11,18,25^ All tests were performed two-sided, and differences were considered statistically significant when *p* < 0.05. Statistical analyses were performed using Statistical Package for Social Sciences (SPSS) version 25.0 (IBM Corporation, Armonk, NY). Kaplan Meyer survival curves were created using R version 4.0.2 (http://www.r-project.org).

### Ethical Considerations

The local Medical Ethics Review Committees approved the collection of data for this study of the Erasmus Medical Center and the Radboud University Medical Center.

## Results

Between March 2010 and October 2020, 394 patients underwent a first CRS-HIPEC procedure for colorectal PM in the Erasmus Medical Center in Rotterdam and the Radboud University Medical Center in Nijmegen. Four patients were excluded, because colorectal PM were not histologically proven in the preoperative workup or at CRS-HIPEC. The median follow-up was 26 months for all survivors (IQR 13–44).

### Baseline and Intraoperative Characteristics, and Postoperative Outcomes

Of 390 patients included in this study, 179 (45.9%) patients had s-PM, whereas 211 (54.1%) patients were stratified as m-PM. Baseline characteristics are displayed in Table 2. s-PM patients presented with higher TN-stages (*p* < 0.001) and more often had a poorly differentiated primary tumor (29.8% vs. 13.7%, *p* = 0.001). Mucinous adenocarcinomas (27.5% vs. 21.1%) and signet ring cell carcinomas (13.7% vs. 3.3%) were more common in the s-PM group (*p* = 0.001). Of the s-PM patients, 78.2% underwent prior colorectal cancer surgery and the primary tumor was resected in 45.8% before CRS-HIPEC. The median time between primary surgery and CRS-HIPEC procedure was 60 days [IQR 28–105] for s-PM patients that underwent prior surgery. These patients were stratified in two groups based on the median time interval of 60 days. For patients with a time interval of 60 days or more, prior surgery was more often performed in an acute setting (29.9% vs. 15.1%, *p* = 0.035), and the primary tumor was more often resected before CRS-HIPEC (68.7% vs. 49.3%, *p* = 0.020). s-PM patients more often received perioperative chemotherapy to CRS-HIPEC (44.5% vs. 27.3%, *p* = 0.001). Intraoperative characteristics and postoperative outcomes are shown in Table 3. Severe complications (Clavien–Dindo grade ≥ III) and reoperations after CRS-HIPEC occurred more often in the m-PM group (29.9% vs. 18.4%, *p* = 0.009; 14.7% vs. 7.3%, *p* = 0.021; respectively). m-PM patients with severe complications were less often treated with adjuvant chemotherapy to CRS-HIPEC (9.7%) than patients who did not experience a severe complication (27.2%, *p* = 0.006). In the s-PM group, the rate of severe complications did not significantly differ between patients who underwent prior colorectal surgery (19.3%) and patients who did not (15.4%, *p* = 0.578).

**Table 2:** Baseline characteristics

	Total *N* = 390	Synchronous *N* = 179 (45.9%)	Metachronous *N* = 211 (54.1%)	*p* value
Gender				
Male	191 (49.0)	85 (47.5)	106 (50.2)	0.588
Female	199 (51.0)	94 (52.5)	105 (49.8)	
Age (year)	64 [55–71]	64 [54–71]	64 [55–71]	0.480
BMI (kg/m^2^)	25.7 [23.1–29]	25.6 [23.0–28.9]	25.8 [23.5–29.1]	0.488
Preoperative CEA	7.9 [3.7–14.0]	7.8 [2.8–19.7]	7.9 [4.4–14.0]	0.665
Smoking (past or current)				
Yes	125 (34.6)	61 (36.3)	64 (33.2)	0.531
No	236 (65.4)	107 (63.7)	129 (66.8)	
*Missing*	29 (7.4)	11 (2.8)	18 (4.6)	
ASA-classification				
1	53 (13.8)	31 (17.6)	22 (10.6)	0.122
2	249 (64.8)	111 (63.1)	138 (66.3)	
≥ 3	82 (21.4)	34 (19.3)	48 (23.1)	
Missing	6 (1.5)	3 (1.7)	3 (1.4)	
Primary tumor location				
Ascending colon	154 (39.5)	74 (41.3)	80 (37.9)	0.059
Transverse colon	30 (7.7)	19 (10.6)	11 (5.2)	
Descending colon	33 (8.5)	9 (5.0)	24 (11.4)	
Sigmoid	121 (31.0)	52 (31.0)	69 (32.7)	
Rectum	52 (13.3)	25 (14.0)	27 (12.8)	
T stage primary tumor				
T1	6 (1.6)	0 (0)	6 (2.9)	< 0.001
T2	12 (3.1)	2 (1.2)	10 (4.8)	
T3	173 (45.3)	59 (34.3)	114 (54.3)	
T4	191 (50.0)	111 (64.5)	80 (38.1)	
Missing	*8 (2.1)*	*7 (3.9)*	*1 (0.5)*	
N stage primary tumor				
N0	90 (23.9)	21 (12.4)	69 (33.5)	< 0.001
N1–N2	286 (76.1)	149 (87.6)	137 (66.5)	
Missing	14 (3.6)	9 (5.0)	5 (2.3)	
Synchronous liver metastases^a^				
Yes	42 (10.8)	21 (11.7)	21 (10.0)	0.572
Differentiation				
Good/moderate	245 (78.5)	106 (70.2)	139 (86.3)	0.001
Poor	67 (21.5)	45 (29.8)	22 (13.7)	
Missing	78 (20.0)	28 (15.6)	50 (23.7)	
Histology				
Adenocarcinoma	205 (67.2)	90 (58.8)	115 (75.7)	0.001
Mucinous adenocarcinoma	74 (24.3)	42 (27.5)	32 (21.1)	
Signet ring cell carcinoma	26 (8.5)	21 (13.7)	5 (3.3)	
Missing	85 (21.8)	26 (14.5)	59 (28.0)	
Prior colorectal cancer surgery				
Yes	351 (90.0)	140 (78.2)	211 (100)	< 0.001
No	39 (10.0)	39 (21.8)	0 (0)	
Prior surgery type				
Acute	67 (19.6)	31 (22.1)	36 (17.9)	0.333
Elective	274 (80.4)	109 (77.9)	165 (82.1)	
Missing	10 (2.6)	0 (0)	10 (4.7)	
Primary tumor status at HIPEC				
In situ	117 (30.0)	97 (54.2)	1 (0.5)	< 0.001
Resected	273 (70.0)	82 (45.8)	210 (99.5)	
Prior chemotherapy				
Yes	104 (26.7)	0 (0)	104 (49.5)	< 0.001
Perioperative chemotherapy ^b^				
Yes	131 (35.3)	77 (44.5)	54 (27.3)	0.001

Continuous variables are shown as median [IQR]. Frequencies are shown as N (%)

*BMI* body mass index; *ASA* American association for anesthesiology; *PM* peritoneal metastasis

^a^Synchronous liver metastases to primary tumor

^b^Neoadjuvant and/or adjuvant chemotherapy around CRS-HIPEC

**Table 3:** Intraoperative characteristics and postoperative outcomes

	Total *N* = 390	Synchronous *N* = 179 (45.9%)	Metachronous = 211 (54.1%)	*p* value
PCI	10 [5–15]	11 [5–15]	9 [5–14]	0.389
CCR-score				
R1	380 (97.4)	174 (97.2)	206 (97.6)	0.417
R2a	4 (1.0)	1 (0.6)	3 (1.4)	
R2b	6 (1.5)	4 (2.2)	2 (0.9)	
Procedure time (min)^a^	375 [304–449]	379 [316–452]	373 [300–449]	0.358
Blood loss (L)^b^	1.0 [0.5–1.6]	1.0 [0.5–1.9]	0.9 [0.6–1.5]	0.434
HIPEC regimen				
MMC	274 (70.3)	118 (65.9)	156 (73.9)	0.085
Oxaliplatin	116 (29.7)	61 (34.1)	55 (26.1)	
Anastomosis				
Yes	231 (60.6)	117 (66.5)	114 (55.6)	0.030
Median number/patient	1 [0–1]	1 [1–1]	1 [0–1]	0.196
Stoma				
Total	144 (36.9)	78 (43.6)	66 (31.3)	0.012
Ileostomy	29 (7.6)	19 (10.6)	10 (4.7)	0.170
Colostomy	115 (30.2)	59 (33.0)	56 (26.5)	
Length of stay (days)	14 [11–18]	14 [11–19]	14 [11–18]	0.798
Complications (any grade)				
Any complication	203 (52.1)	94 (52.5)	109 (51.7)	0.866
Anastomotic leakage	24 (6.2)	7 (3.9)	17 (8.2)	0.081
Postoperative hemorrhage	17 (4.4)	5 (2.8)	12 (5.8)	0.154
Intra-abdominal abscess	30 (7.8)	10 (5.6)	20 (9.7)	0.136
Ileus/gastroparesis^d^	57 (14.6)	30 (16.8)	27 (12.8)	0.270
Wound complications	35 (9.0)	17 (9.5)	18 (8.5)	0.739
Pneumonia	24 (6.2)	13 (7.3)	11 (5.3)	0.429
Pulmonary embolism	5 (1.3)	3 (1.7)	2 (1.0)	0.539
Cardiac complications	24 (6.2)	10 (5.6)	14 (6.8)	0.633
UTI	28 (7.3)	14 (7.8)	14 (6.8)	0.689
Complications Clavien–Dindo ≥ III^e^	96 (24.6)	33 (18.4)	63 (29.9)	0.009
Reoperations	44 (11.3)	13 (7.3)	31 (14.7)	0.021
Clavien–Dindo grade				
I	36 (9.2)	19 (10.6)	17 (8.1)	0.148
II	85 (21.8)	47 (26.3)	38 (18.0)	
IIIa	47 (12.1)	18 (10.1)	29 (13.7)	
IIIb	31 (7.9)	11 (6.1)	20 (9.5)	
IVa	11 (2.8)	2 (1.1)	9 (4.3)	
IVb	1 (0.3)	0 (0)	1 (0.5)	
V	6 (1.5)	2 (1.1)	4 (1.9)	

Continuous variables are shown as median [IQR]. Frequencies are shown as *N* (%)

*PCI* Peritoneal Cancer Index; *CCR* completeness of cytoreduction; *MMC* mitomycin-C

^a^Procedure time was available for 371 patients

^b^Blood loss data was available for 370 patients

^c^Anastomosis data was available for 381 patients

^d^Ileus (*n* = 16), gastroparesis (*n* = 45)

^e^Clavien–Dindo classification ≥ III (i.e., reintervention, extended ICU stay/readmission to ICU, or treatment-related death)

### Disease-Free Survival

**Fig. 1:**
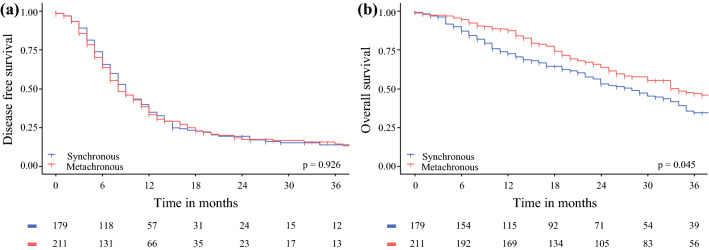
Kaplan–Meier survival curves for disease-free survival (**A**) and overall survival (**B**) for patients with synchronous onset versus metachronous onset of peritoneal metastasis. The log-rank *p*-values are displayed in the bottom right corner

A total of 287 patients (77.2%) had a recurrence of disease during follow-up. Of these patients, 108 (37.6%) had peritoneal recurrence, 78 patients (27.2%) had systemic recurrence, and 101 patients (35.2%) had local as well as systemic recurrence of disease. The location of recurrence of disease did not significantly differ between the s-PM and m-PM groups (*p* = 0.627). The median DFS for all patients was 8 months. For the s-PM patients, the median DFS was 9 months, compared with 8 months for the m-PM patients (*p* = 0.962; Fig. 1a). Multivariable analysis showed that age (HR 0.99, 95% CI 0.97–1.00, *p* = 0.035) and PCI (1.05, 95% CI 1.02–1.07, *p* = 0.001) were independently associated with DFS (Table 4).

**Table 4:** Cox proportional regression analysis for predictors of DFS

	Univariate analysis HR (95% CI)	*p* value	Multivariate analysis HR (95% CI)	*p* value
Onset of PM				
Synchronous	1			
Metachronous	1.01 (0.80–1.27)	0.929		
Gender				
Male	1			
Female	1.02 (0.81–1.29)	0.860		
Age (years)	0.99 (0.98–1.00)	0.238	0.99 (0.97–1.00)	0.035
ASA-classification				
1	1		1	
2	1.33 (0.95–1.87)	0.099	1.44 (0.91–2.29)	0.118
≥ 3	1.19 (0.79–1.79)	0.406	1.64 (0.97–2.77)	0.066
Primary tumor location				
Ascending colon	1			
Transverse colon	1.27 (0.82–1.97)	0.291		
Descending colon	1.19 (0.77–1.83)	0.444		
Sigmoid	1.19 (0.90–1.58)	0.227		
Rectum	1.27 (0.89–1.82)	0.193		
N stage primary tumor				
N0	1		1	
N1–2	1.24 (0.93–1.65)	0.142	1.37 (0.95–1.99)	0.092
Differentiation				
Good/moderate	1			
Poor	1.36 (1.00–1.85)	0.051		
Histology				
Adenocarcinoma	1			
Mucinous adenocarcinoma	0.96 (0.71–1.30)	0.790		
Signet ring cell carcinoma	1.18 (0.72–1.92)	0.519		
PCI	1.05 (1.03–1.07)	< 0.001	1.05 (1.02–1.07)	0.001
CCR-score				
R1	1			
≥ R2a	1.60 (0.66–3.89)	0.300		
Complications				
Clavien–Dindo ≥ III	1.26 (0.96–1.65)	0.099		
Perioperative chemotherapy				
Yes	0.76 (0.60–0.97)	0.030	0.80 (0.58–1.09)	0.158

*PM* peritoneal metastasis; *ASA* American association of anesthesiology; *PCI* peritoneal cancer index; *CCR* completeness of cytoreduction

### Overall Survival

Median OS for all patients was 32 months, and during follow-up 215 patients deceased. Median OS was significantly shorter for s-PM (28 months) compared to m-PM patients (33 months, *p* = 0.045; Fig. 1b). In multivariable analysis, the onset of PM was not associated with OS (*p* = 0.193). Factors that were independently associated with OS in multivariable analysis were N stage (HR 1.76, 95% CI 1.9–2.84, *p* = 0.020) and poor differentiation of the primary tumor (HR 1.95, 95% CI 1.32–2.90), as well as PCI (HR 1.07, 95% CI 1.03–1.10, *p* < 0.001; Table 5).

**Table 5:** Cox proportional regression analysis for predictors of OS

	Univariate analysis HR (95% CI)	*p* value	Multivariate analysis HR (95% CI)	*p* value
Onset of PM				
Synchronous	1			
Metachronous	0.76 (0.58– 1.00)	0.048		
Gender				
Male	1			
Female	1.00 (0.76–1.30)	0.976		
Age (years)	1.01 (1.00–1.02)	0.167	1.02 (1.00–1.03)	0.099
ASA-classification				
1	1			
2	1.22 (0.82–1.80)	0.322		
≥3	1.17 (0.73–1.87)	0.526		
Primary tumor location				
Ascending colon	1			
Transverse colon	1.16 (0.69–1.94)	0.583		
Descending colon	0.95 (0.57–1.60)	0.858		
Sigmoid	0.86 (0.62–1.20)	0.388		
Rectum	1.17 (0.79–1.73)	0.429		
N stage primary tumor				
N0	1		1	
N1–2	1.44 (1.03–2.02)	0.034	1.76 (1.9–2.84)	0.020
Differentiation				
Good/moderate	1		1	
Poor	2.09 (1.50–2.92)	< 0.001	1.95 (1.32–2.90)	0.001
Histology				
Adenocarcinoma	1			
Mucinous adenocarcinoma	1.09 (0.76–1.56)	0.648		
Signet ring cell carcinoma	2.79 (1.74–4.48)	< 0.001		
PCI	1.07 (1.05–1.01)	< 0.001	1.07 (1.03–1.10)	< 0.001
CCR-score				
R1	1			
≥ R2a	3.26 (1.66–6.37)	0.001		
Complications				
Clavien–Dindo ≥ III	1.45 (1.08–1.96)	0.015	1.40 (0.94–2.09)	0.097
Perioperative chemotherapy				
Yes	0.81 (0.61–1.08)	0.151		

*PM* peritoneal metastasis; *ASA* American Association of Anesthesiology; *PCI* peritoneal cancer index; *CCR* completeness of cytoreduction

### Disease-Free Interval

The median disease-free interval (DFI) between the diagnosis of the primary tumor and PM was 19 months [IQR 11–30] for m-PM patients. DFI was not associated with DFS (HR 1.00, 95% CI 0.99–1.01, *p* = 0.375) or OS (HR 1.00, 95% CI 0.99–1.01, *p* = 0.974) in these patients. Median DFS was 8 months [IQR 5–17] for m-PM patients with a short DFI compared with 9 months [IQR 5–17] for patients with a long DFI (*p* = 0.660). Regarding OS, the median was 40 months [IQR 15–NR] for the patients with a short DFI versus 33 months [IQR 20–52] for the patients with a long DFI (*p* = 0.747).

## Discussion

This study showed that patients with synchronous colorectal peritoneal metastasis (s-PM) had impaired overall survival compared with patients with metachronous PM (m-PM). However, this is probably explained by other factors, as synchronous onset of PM was not identified as an independent predictor of OS in multivariable analysis. Disease-free survival (DFS) did not differ between s-PM and m-PM patients.

Because of considerable morbidity after CRS-HIPEC for colorectal PM (24.6% in the current cohort), the identification of prognostic factors for optimal patient selection is needed. Some previous studies proposed that the timing of onset of metastases from CRC could be of prognostic value.^14–17^ Few studies investigated the impact of synchronous onset in patients with colorectal PM undergoing CRS-HIPEC, and conflicting results were published (Table 1).^1,12,18–20^

A study by Hentzen et al. in the Netherlands reported a decreased DFS, but not OS, in m-PM patients after CRS-HIPEC.^19^ This is the opposite of the hypothesis that synchronous onset would predict poor survival. This might partially be explained by the use of perioperative chemotherapy. In the study by Hentzen et al., s-PM patients were treated more often with perioperative systemic chemotherapy around CRS-HIPEC than m-PM patients. They also reported that perioperative chemotherapy was associated with longer DFS, but not OS. In the current cohort, the use of perioperative chemotherapy was associated with longer DFS in univariable, but not in multivariable analysis. Because currently there is no consensus in the field regarding the use of perioperative systemic chemotherapy around CRS-HIPEC, the CAIRO-6 trial was initiated.^26,27^ In this ongoing, randomized, controlled trial in the Netherlands, perioperative systemic therapy and CRS-HIPEC is compared to CRS-HIPEC alone. Hopefully, this trial will give clarity about the role of perioperative chemotherapy.

In the current cohort, s-PM patients were also more commonly treated with perioperative chemotherapy, but this difference was smaller than for the cohort of Hentzen et al. The difference in treatment regimen between s-PM and m-PM patients is partially explained by the (intended) treatment of the primary tumor. For some s-PM patients, PM was diagnosed at the (intended) resection of the primary tumor. Some of these patients had received neoadjuvant systemic therapy. This treatment was classified as neoadjuvant therapy to the completion surgery consisting of CRS-HIPEC. Another explanation is the difference in adjuvant treatment to CRS-HIPEC. The rate of severe complications after CRS-HIPEC was higher in the m-PM group, and these patients were less likely to receive adjuvant chemotherapy.

Hentzen et al. reported a remarkably longer median DFS (15 months for s-PM vs. 11 months for m-PM patients) than the current study (9 months for s-PM vs. 8 months for m-PM patients), while median OS was comparable. A recent population-based study by Bakkers et al. in the Netherlands reported an even longer DFS (14.1 months for s-PM vs. 21.5 months for m-PM patients).^1^ An explanation for the difference in DFS might be that there is no nationwide protocol for follow-up after CRS-HIPEC. In the cohort of Hentzen et al., CT scans were only performed when recurrence was suspected (e.g., clinical symptoms or increasing CEA levels). CT scans were performed every 6 months during the first 2 years of follow-up in the current study. This might have led to earlier detection of recurrence, resulting in a difference in DFS, but not OS. It is debatable whether earlier detection of recurrence after CRS-HIPEC is preferable because the curative options for recurrence after CRS-HIPEC are limited.

A study by Wong et al. described the same follow-up protocol as the current study and reported a similar median DFS (9.5 months) in 102 patients who underwent CRS-HIPEC from 2003 to 2018.^20^ Corresponding to the findings of the current study, Wong et al. reported an impaired OS, but not DFS, in s-PM patients. In addition, synchronous onset of PM could not be identified as an independent predictor of OS. This is in line with two previous meta-analyses reporting on prognostic factors after CRS-HIPEC and the study by Bakkers et al.^1,12,18^ This suggests that s-PM is probably not a predictor of early recurrence but that it illustrates poor tumor characteristics and a more advanced disease. At baseline, s-PM patients had higher TN-stages, poor differentiation and signet cell ring histology of the primary tumor. These factors, which are associated with poor tumor characteristics and more advanced disease, independently predict survival.^7,11,18^ This probably explains why s-PM patients had impaired OS, but synchronous onset was not significantly associated with OS in multivariable analysis.

For patients with metachronous onset of PM, the time interval between diagnosis of the primary tumor and the diagnosis of PM (DFI) was not associated with DFS, nor with OS. This supports the hypothesis that the time of onset of PM is not an independent prognostic factor*.*

In the current cohort, lymph node metastasis, poor primary tumor differentiation, and PCI were independently associated with poorer OS. Signet cell ring histology also was associated with OS in univariable but not in multivariable analysis.

As CRS-HIPEC was more often the primary treatment for s-PM patients, bowel resections, and the creation of an anastomosis and/or stoma were more often performed in this group. However, significantly more severe complications (i.e., Clavien-Dindo ≥ III) and reoperations after CRS-HIPEC were reported in m-PM patients. m-PM patients more often underwent prior colorectal cancer surgery, with a longer time interval between primary surgery and CRS-HIPEC. Several previous studies showed that (extensive) prior surgery is a risk factor for the occurrence of complications after CRS-HIPEC.^28,29^ These studies did not report on the time interval between prior surgery and CRS-HIPEC. In the current cohort, a substantial number of s-PM patients also underwent prior colorectal cancer surgery (78%). The primary tumor was resected before CRS-HIPEC for almost half of the s-PM patients, reflecting extensive surgery. Prior surgery often is performed in the referring center and in an acute setting, resulting in a considerable time interval (median 60 days) between primary surgery and completion surgery consisting of CRS-HIPEC. Contrary to the aforementioned hypothesis, the rate of severe postoperative complications was not higher for s-PM patients that underwent prior colorectal cancer surgery. Hence, the time interval between prior surgery and CRS-HIPEC seems to play a role in the risk of postoperative complications. Previous studies showed that postoperative complications were associated with impaired survival after CRS-HIPEC.^25,30^ In the current study, severe postoperative complications (Clavien-Dindo ≥ III) were associated with poorer OS in univariate but not in multivariate analysis. This is probably explained by the association of postoperative complications with higher PCI, reflecting extensive surgery.

PCI was the only variable that was independently associated with both DFS and OS. PCI is preferably determined by laparoscopy, or if not possible, by radiological imaging. However, preoperative underestimation of PCI is not uncommon.^13^ To improve patient selection, future research should focus on improving preoperative prediction of PCI. A currently ongoing study in the Netherlands, the DISCO-trial, was initiated to determine the role of MRI in detecting colorectal PM in patients who are considered for CRS-HIPEC. In this multicenter, randomized study, a diagnostic workup with MRI is compared to the standard workup with surgical staging. The results of this study will hopefully contribute to improved preoperative PCI estimation.

### Limitations

This study was mainly limited by its retrospective nature, which could have resulted in selection bias. Patients with a high PCI (i.e., 20 or higher) were not eligible for CRS-HIPEC and were thus not included in this study. Patients with aggressive synchronous PM probably present with higher PCI and could therefore have been excluded. The study by Bakkers et al. showed that s-PM patients were less often treated with CRS-HIPEC than m-PM patients.^1^ s-PM patients might have worse survival outcomes in the general population of patients with colorectal PM but not in this selected cohort of patients undergoing CRS-HIPEC. Another limitation of the current study was the relatively short follow-up for surviving patients. Therefore, we presented the 3-year survival data.

## Conclusions

The current study showed that synchronous onset of colorectal PM was associated with impaired overall survival, but probably due to confounding factors associated with poor tumor characteristics and advanced disease. Tumor differentiation, lymph node status, and PCI are more valuable predictors for survival after CRS-HIPEC and are important factors that could aid in shared decision making.

## Race/Ethnicity Data

The race/ethnicity distribution was not reported as this data was not collected in the database that was analyzed for this study. Due to the retrospective nature of this study, this data could not be obtained.
